# Comparative Analysis of Sonication, Microfluidics, and High-Turbulence Microreactors for the Fabrication and Scaling-Up of Diclofenac-Loaded Liposomes

**DOI:** 10.3390/pharmaceutics18010105

**Published:** 2026-01-13

**Authors:** Iria Naveira-Souto, Roger Fabrega Alsina, Elisabet Rosell-Vives, Eloy Pena-Rodríguez, Francisco Fernandez-Campos, Jessica Malavia, Xavier Julia Camprodon, Maximilian Schelden, Nazende Günday-Türeli, Andrés Cruz-Conesa, Maria Lajarin-Reinares

**Affiliations:** 1 Department of Biochemistry and Physiology, School of Pharmacy and Food Sciences, University of Barcelona, 08028 Barcelona, Spain; 2 Pharmaceutical Innovation, Reig Jofre Laboratories, 08970 Barcelona, Spain; elisabet.rosell@upf.edu (E.R.-V.); eloy.penarodriguez@microcaps.ch (E.P.-R.); francisco.fernandez@labiana.com (F.F.-C.); xjulia@reigjofre.com (X.J.C.); mlajarin@reigjofre.com (M.L.-R.); 3 Pharmaceutical Development, Reig Jofre Laboratories, 08970 Barcelona, Spain; rfabrega@reigjofre.com (R.F.A.); jmalavia@reigjofre.com (J.M.); 4 Nanobioengineering, Institute for Bioengineering of Catalonia (IBEC), 08028 Barcelona, Spain; 5 Doctorate Program in Biomedicine, University of Barcelona, 08036 Barcelona, Spain; 6 MyBiotech GmbH, Industriestr. 1b, 66802 Überherrn, Germany; m.schelden@mybiotech.de (M.S.); n.guenday-tuereli@mybiotech.de (N.G.-T.); 7 IRIS Technology Solutions, 08940 Barcelona, Spain; acruz@iris-eng.com

**Keywords:** diclofenac, liposomes, SUVs, encapsulation, sonication, microfluidics, microreactors, scale-up, design of experiments

## Abstract

**Background**: Liposomes are attractive topical carriers, yet translating laboratory fabrication to scalable, well-controlled processes remains challenging. **Objectives**: We compared three manufacturing methods for diclofenac-loaded liposomes: probe sonication, microfluidic mixing, and a high-turbulence microreactor, under a Quality-by-Design framework. **Methods**: Differential scanning calorimetry (DSC) was used to define a processing-relevant liquid-crystalline temperature window for the lipid excipients. For sonication scale-up, a Plackett-Burman screening design identified key process factors and supported an energy-density (W·s·L^−1^) control approach. For microfluidics, the effects of flow-rate ratio (FRR) and total flow rate (TFR) were mapped and optimized using a desirability function. Microreactor trials were performed at elevated throughput. Residual ethanol during post-processing was monitored at-line by Raman spectroscopy calibrated against gas chromatography (GC). Particle size and dispersity were measured by DLS and morphology assessed by cryo-TEM. **Results**: DSC supported a 70–85 °C processing window. Sonication scale-up using an energy-density target (~11,000 W·s·L^−1^) reproduced lab-scale quality at 8 L (Z-average ~87–92 nm; PDI 0.16–0.23; %EE 86–94%). Microfluidics optimization selected FRR 3:1/TFR 4 mL·min^−1^, yielding ~64 nm liposomes with PDI ~0.13 and %EE ~93%. The microreactor achieved ~50 nm liposomes with %EE ~95% at 50 mL·min^−1^. Cryo-TEM corroborated size trends and showed no evident aggregates. **Conclusions**: All three routes met topical CQAs (~50–100 nm; PDI ≤ 0.30; high %EE). Method selection should be guided by target size/dispersity and operational constraints: sonication enables energy-based scale-up, microfluidics offers precise size control, and microreactors provide higher throughput.

## 1. Introduction

Liposomes are nanoscale vesicles, typically ranging from 20 to 400 nm for unilamellar vesicles, composed of phospholipid bilayers that have garnered extensive interest in pharmaceutical, cosmetic, and nutraceutical applications. Their unique structure allows for the encapsulation of both hydrophilic molecules within the aqueous core and lipophilic compounds within the lipid bilayer, offering versatility in delivering a wide range of active agents [1]. This dual encapsulation capability, combined with their biocompatibility and ability to enhance the bioavailability and stability of encapsulated compounds, has made liposomes a key platform in controlled release systems [2,3,4,5]. Despite their potential, the fabrication of liposomes remains a critical challenge, particularly in achieving reproducibility, size uniformity, and scalability—factors essential for their translation from laboratory research to industrial-scale production [2,3,4,6].

The bilayer phase state critically governs liposome assembly and drug loading. In the gel (Lβ′) state, phospholipid acyl chains are ordered and tightly packed, yielding rigid membranes with low lateral diffusion and limited propensity for membrane bending and closure, which hinder vesicle formation and reduce permeability to solutes. Upon transition conditions pertaining to the liquid crystalline (Lα) state, chain disorder, and lateral mobility increase, facilitating lamellar reorganization, vesicle closure, and the incorporation of co-lipids (e.g., cholesterol) and amphiphiles; this typically improves processability, narrows size distributions, and supports higher and more reproducible encapsulation. Consequently, processing conditions are commonly referenced to the main phase transition of the bulk phospholipids and its modulation by composition (e.g., hydrogenation degree and cholesterol content), with the goal of ensuring sufficient bilayer fluidity during formation while avoiding unnecessary thermal stress [7,8].

**Figure 1:**
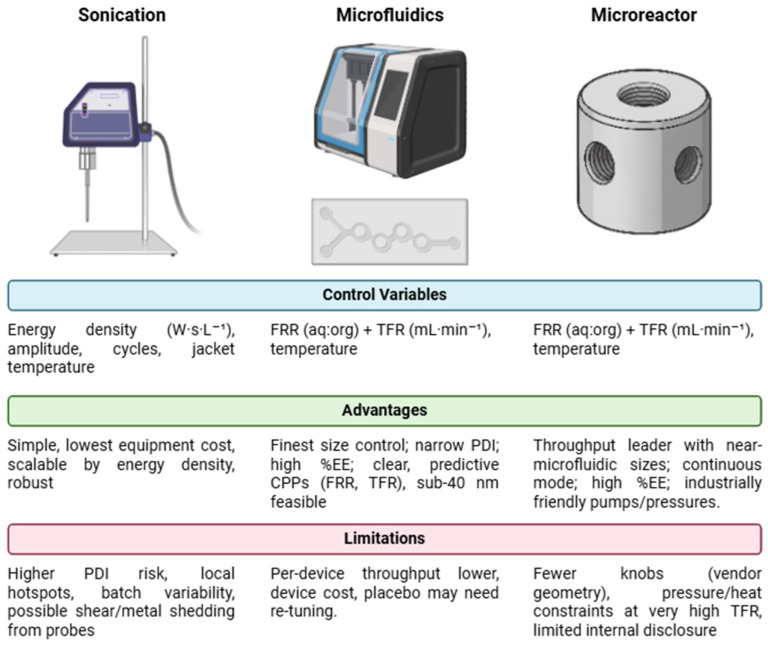
Comparative manufacturing of liposomes: sonication, microfluidics, and microreactors. Created in BioRender. Naveira Souto, I. (2026) [18].

Among the numerous fabrication methods available, probe sonication, microfluidics, and high-turbulence microreactors represent complementary strategies with distinct advantages and trade-offs (Figure 1). Sonication, one of the most traditional approaches, is valued for its simplicity, broad availability, and low capital cost, making it a popular choice for laboratory-scale liposome production. Control over critical quality attributes (CQAs), however, notably size and polydispersity, is comparatively coarse because vesicle formation is governed by cavitation events that are sensitive to amplitude, duty cycle, and heat generation. Localized thermal and mechanical stresses can also challenge heat- or shear-sensitive cargos, and scale-up often requires explicit normalization to energy density to preserve product attributes [2,3,4,6,9].

In contrast, microfluidic mixing, particularly hydrodynamic focusing/solvent displacement in confined channels, represents a more advanced and innovative approach to liposome fabrication. This method leverages the precise manipulation of fluids at the microscale to achieve consistent and reproducible particle sizes, resulting in liposomes with superior uniformity and narrower size distributions, within the defined operating windows of the flow rate ratio (FRR) and total flow rate (TFR). Microfluidics also allow for greater control over the encapsulation process, making it highly advantageous for applications requiring stringent quality standards. Nevertheless, the method poses challenges related to the cost of equipment, technical complexity, and the need to optimize scaling parameters to make it viable for large-scale production, although practical translation is supported by platform instruments that permit bench-to-pilot scaling via parallelization or larger cartridges, a with per-device throughputs that are modest compared with bulk processes [6,10,11,12,13].

A third technology for the production of liposomes employs the use of microreactors, e.g., those used in this study (MyBiotech, Überherrn, Germany), which generate highly turbulent flow conditions and high-velocity mixing under strictly controlled process conditions. While providing comparable results to microfluidic devices (i.e., reproducible low particle sizes achieved with high control), microreactors can deliver considerably higher volumetric throughputs up to the multiple L·min^−1^ range, offering excellent scale-up capabilities and high reproducibility. Operated at low to medium pressures ranging, dependent on the application, from 10 to 400 bar, they allow the use of high-throughput gear or membrane pumps and avoid the requirement of external cooling and expensive high-pressure equipment. These represent substantial advantages over common high-pressure technologies like valve homogenizers or microfluidizers, which are usually operated at energy intensive pressures of 1000–2000 bar [14,15,16,17].

The selection of an appropriate fabrication method significantly impacts the physicochemical properties of liposomes, including their size, polydispersity index (PDI), encapsulation efficiency, and stability. These properties, in turn, influence their performance and suitability for specific applications, from drug delivery systems to cosmetic formulations [2,3,4,5,19]. In this study, we focus on liposomes designed to encapsulate diclofenac, a widely used nonsteroidal anti-inflammatory drug (NSAID), that is commonly employed in the treatment of pain and inflammation but is associated with gastrointestinal side effects and limited bioavailability [20]. Accordingly, we propose diclofenac-loaded liposomes for topical administration, concentrating the drug at the site of action while limiting systemic exposure, thereby reducing off-target effects and supporting targeted, local therapy. Encapsulation within liposomes offers a promising strategy to mitigate these limitations by enhancing its stability, targeting delivery, and potentially reducing systemic toxicity [21,22,23].

Scalability remains a critical consideration when transitioning from laboratory-scale experiments to industrial production [2,6,24,25]. While sonication has been widely used in research settings, its scalability is often limited [24,25]. Microfluidics, although promising for high-quality production, requires further investigation into its feasibility for larger volumes and industrial applications [6,12,26]. In this context, high-turbulence microreactors (e.g., impinging designs) provide a complementary route that retains the microfluidic-like control of size while delivering substantially higher throughputs in a continuous mode—an attractive path in terms of industrial translation, that is contingent on establishing operating maps (e.g., FRR at elevated TFR) and demonstrating long-run robustness [14,15,16,17].

This study aims to conduct a comprehensive comparison of three manufacturing routes for diclofenac-loaded liposomes: sonication (lab optimization and pilot scale with energy-normalized control), microfluidic mixing (flow rate ratio, FRR, total flow rate, TFR, and screening with multi-response optimization), and a high-turbulence microreactor operated at elevated throughputs. Specifically, we evaluate the efficiency of each route and the quality of the resulting liposomes in terms of hydrodynamic size (Z-average), polydispersity index (PDI), encapsulation efficiency (%EE), and zeta potential, corroborated by cryo-TEM. We also examine scalability via a Plackett–Burman design and energy density modeling for sonication, delineate an FRR/TFR operating window for microfluidics with experimental confirmation, and identify a practical operating point for the microreactor.

By articulating the control levers intrinsic to each route (energy density for sonication, FRR/TFR for microfluidics, and FRR at elevated TFR for the microreactor), this work clarifies the trade-offs between product quality (size/dispersity, %EE, and colloidal stability) and operational performance (throughput, hardware complexity, and ease of scale-up). The resulting evidence base is intended to guide researchers and industry practitioners in selecting the most suitable manufacturing strategy for topical diclofenac liposomes, balancing quality, efficiency, and scalability under a Quality-by-Design framework.

## 2. Materials and Methods

### 2.1. Materials

Phosphatidylcholine and phosphatidylcholine hydrogenated (Lipoid, Ludwigshafen, Germany), Tween 80 (Croda Iberica S.A., Barcelona, Spain), α-Tocopherol (Merck Life Science, Barcelona, Spain), cholesterol (Carbogen Amcis, Bubendorf, Switzerland), sodium diclofenac (DCF) (Azelis Spain S.A., Barcelona, Spain), ethanol 96° (Alcoholes Oliva S.A., Barcelona, Spain), and purified water (in-house, Milli-Q^®^, 18.2 MΩ·cm; Millipore, Burlington, MA, USA) were used to formulate the liposomes. Phosphate-buffered saline (PBS, Ph 7.4, 10 mM phosphate, Thermo Fisher Scientific, Waltham, MA, USA) was used as the aqueous phase. Sodium nipagin and sodium nipasol (Lemmel S.A., Barcelona, Spain) were used as formulation preservatives.

Methanol (HPLC grade) and buffer salts (analytical grade) for chromatography were from Merck KGaA (Darmstadt, Germany). All reagents were used as received without further purification unless otherwise stated.

### 2.2. Work Temperature Determination

Differential scanning calorimetry (DSC Q20, TA Instruments, New Castle, DE, USA) was used to analyze the thermal transitions of phosphatidylcholine, hydrogenated phosphatidylcholine, and cholesterol. Approximately 3–5 mg of each material was sealed in aluminum pans and scanned at 10 °C·min^−1^ over 20–200 °C. Instrument temperature and enthalpy calibration were performed according to the manufacture’s recommendations prior to analysis. DSC was used here as a supportive tool to identify the main transition region relevant for selecting the processing temperature window. The thermograms shown correspond to representative scans obtained from fresh material lots; transition temperatures were verified to be consistent across material lots. The working temperature window for processing (Section 3.1) was selected based on the main gel-to-liquid crystalline transitions.

### 2.3. Preparation of Liposomes by Sonication (Lab Scale)

Phosphatidylcholine (0.73% *w*/*w*), hydrogenated phosphatidylcholine (0.20% *w*/*w*), Tween 80 (0.05% *w*/*w*), α-tocopherol (0.01% *w*/*w*), cholesterol (0.01% *w*/*w*), sodium diclofenac (0.50% *w*/*w*), and ethanol (10% *w*/*w*) were mixed to prepare the organic phase [27]. DCF was dissolved in the organic solution; for the placebo formulation, DCF was not added. The organic solution and phosphate buffer (85.505% *w*/*w*) were preheated to 75 °C. The aqueous phase was added to the organic, and the system was vortex-mixed for 1 min. The mixture was then probe-sonicated (UP400st, Hielscher Ultrasonics, Teltow, Germany, 20% amplitude, 15 s, cumulative energy = 2000 W·s, 25 °C). Placebo liposomes (drug-free liposomes) were processed identically except for the sonication time, which was 40 s at 20% amplitude to reach a comparable energy input. A preservative solution of sodium nipagin (0.20% *w*/*w*) and sodium nipasol (0.02% *w*/*w*) in phosphate buffer (q.s. to 100% *w*/*w*) was added, and the liposomal suspension was allowed to equilibrate at room temperature. All percentages are *w*/*w* relative to the final batch mass.

### 2.4. Scale-Up of Liposomes by Sonication

A Plackett–Burman screening design of experiments with class III resolution using Minitab (Minitab 17 Statistical Software) was employed to maximize the information pertaining to scale-up while minimizing the number of batches. The design comprised eleven runs and four factors, with a significance level of α = 0.05. The responses targeted for optimization were Z-average and PDI [28].

The selected factors were sonication Amplitude (%), Batch Volume (L), the number of Sonication Cycles, and the flow cell Jacket Temperature (°C). Amplitude and the number of Sonication Cycles modulate cavitation energy input, whereas the Jacket Temperature controls thermal stability during processing (kept within the 70–85 °C window defined by DSC; see Section 3.1). Batch Volume was included explicitly to prove scalability [28].

Table 1 summarizes the factor levels for the Plackett–Burman design. To decouple the scale-up effects from API-specific interactions and to limit API consumption during screening, placebo liposomes (i.e., without DCF) were employed in this DoE. Subsequent confirmatory batches at the selected operating window were prepared with DCF for verification (see Section 3.2.3) [28].

Sonication was performed with a UIP1000HdT ultrasonic processor (Hielscher Ultrasonics, Teltow, Germany) equipped with a flow cell, using the specified amplitude settings and cycling regimen. For energy-based analyses, cavitation energy (W·s) was recorded from the sonicator output and normalized by batch volume (W·s·L^−1^) [28].

### 2.5. Preparation of Liposomes by Microfluidics

Liposomes were produced on a NanoAssemblr^®^ Ignite™ (Precision Nanosystems, Vancouver, BC, Canada) using a toroidal micromixer (TrM). The organic and aqueous phases (PBS, pH 7.4) were prepared as in Section 2.3 and preheated to 75 °C. DCF was dissolved in the organic solution; placebo liposomes were prepared without DCF. The flow rate ratio (FRR) was varied between 3:1 and 7:1 (aqueous:solvent ratio) and the total flow rate (TFR) between 4 and 8 mL·min^−1^. Post-chip, the dispersions were immediately diluted with PBS to adjust the organic-solvent fraction to 10% (*w*/*w*).

### 2.6. Screening of Microfluidic Variables

The formulation properties are affected by various experimental parameters, including the ratio between the aqueous and organic phases (flow rate ratio, FRR) and the total formulation flow rate (total flow rate, TFR). To assess these factors, preliminary screening trials were conducted.

A total of 13 batches (n = 13) were produced to evaluate the impact of FRR (3:1–7:1) and TFR (4–8 mL·min^−1^) on Z-average, PDI, and %EE (Table 2). A Definitive Screening Design was employed to analyze both the main and interaction effects of these variables on the responses, using a significance level (α) of 0.05. Model selection was based on statistical criteria, including the coefficient of determination (R^2^), adjusted R^2^, and the Akaike Information Criterion (AIC). Additionally, the order in which the formulations were produced was randomized to reduce the potential impact of external factors on the results.

Statistical analyses of the studied variables were performed using Minitab 17 (Minitab, Inc., State College, PA, USA) to derive the mathematical equations for each model.

Multi-response optimization was then performed using a Derringer–Suich desirability function with equal weights (w = 1) and the following goals: minimize PDI (upper bound 0.20), target size 75 nm (acceptable range 50–100 nm), and maximize %EE (lower bound 85%). The feasible region was constrained to FRR 3:1–7:1 and TFR 4–8 mL·min^−1^ at 75 °C and fixed composition. The operating point with the highest composite desirability (≥0.50) and predicted responses within the pre-specified specification limits was selected. Finally, the set point was experimentally confirmed by manufacturing independent batches (n = 3). The acceptance criteria were as follows: the observed means fall within the model 95% confidence intervals for each response and the relative bias for size and %EE is ≤10% (PDI assessed in absolute units with an acceptable deviation ≤ 0.05).

### 2.7. Scale-Up of Liposomes by Microreactors

The production of liposomes using MyBiotech microreactors was carried out under identical phase compositions and similar flow rate ratios (FRR) as used for small-scale microfluidics production, but with higher total flow rates (TFR). Briefly, organic and aqueous phases were prepared as stated above (Section 2.3. Preparation of Liposomes by Sonication). The aqueous buffer used in all studies was PBS, pH 7.4. Both solutions were heated in a water bath at 75 °C. Experimental trials were carried out using the smallest microreactor configuration available, employing a 100 µm nozzle microreactor system (MyBiotech) with two Knauer HPLC pumps, model P4.1S (KNAUER Wissenschaftliche Geräte GmbH, Berlin, Germany), operated at a FRR of 4:1 (aqueous:solvent ratio) and TFR of 50 mL·min^−1^, with both streams preheated; the outlet was collected isothermally.

### 2.8. Physicochemical Characterization

#### 2.8.1. Particle Size, PDI, and Zeta Potential

Dynamic light scattering (DLS) measurements were performed using a Zetasizer Nano ZS (Malvern Panalytical, Malvern, UK) to determine the Z-average, PDI, and Z-pot of the nanoparticles. Before the measurements, the nanoparticles were diluted as follows: a 1:10 dilution in Milli-Q^®^ water was prepared for determining the Z-average and PDI (n = three measurements, ten runs per measurement), while a 1:50 dilution in PBS was prepared for assessing the zeta potential (n = three measurements, twenty runs per measurement). The measurements were conducted at 25 °C. Unless otherwise stated, data are reported as mean ± standard deviation (SD). The number of independent batches is specified in the corresponding Results tables.

#### 2.8.2. Cryogenic Transmission Electron Microscopy

Cryogenic transmission electron microscopy (cryo-TEM) was used to assess nanoparticle size and morphology. A JEOL JEM-1010 microscope (JEOL, Tokyo, Japan) was employed. Briefly, 1.5 μL of sample was deposited onto the carbon film of a glow-discharged Lacey Carbon 300-mesh copper grid (Ted Pella, Inc., Redding, CA, USA). The grids were prepared in a Vitrobot Mark III (FEI, Eindhoven, The Netherlands) at 100% relative humidity. Excess liquid was blotted with filter paper and the samples were vitrified by plunge-freezing in liquid ethane. Vitrified grids were transferred under cryogenic conditions to a Tecnai F20 electron microscope (FEI, Eindhoven, The Netherlands) at the Cryomicroscopy Unit of the Scientific and Technological Centers of the University of Barcelona using a cryo-holder (Gatan, Inc., Pleasanton, CA, USA). The imaging was performed at 200 kV under low-dose conditions, and the micrographs were recorded with a 4096 × 4096-pixel Eagle CCD camera (FEI, Eindhoven, The Netherlands).

#### 2.8.3. High-Performance Liquid Chromatography (HPLC)

The encapsulation efficiency (%EE) of diclofenac (DCF) in the liposomes was assessed indirectly using Equation (1).

(1) $$\%\;EE=\frac{w_{T}-w_{NE}}{w_{T}}\times 100$$

where w_T_ is the total DCF mass in the formulation and w_NE_ is the non-encapsulated DCF mass in the filtrate.

To briefly summarize, liposomes underwent centrifugation using a 30 kDa Amicon ultracentrifugal filter (Merck Millipore, Billerica, MA, USA) at 4500 rpm for 20 min. Subsequently, the concentration of DCF in both the filtrate and the liposomes was determined employing HPLC (Waters 2695, Barcelona, Spain) equipped with a photodiode array detector (Waters 2996, Barcelona, Spain). The chromatographic column employed was C18 4µ (15 × 0.39 mm) with a particle size of 5 µm. The mobile phase consisted of an isocratic mixture of buffer of pH 7.0 and methanol (35:65). The flow rate was set at 1.0 mL/min, a 10 µL injection volume was used, and quantification was at 276 nm (PDA monitored 190–400 nm). The approximate retention time was 2.5 min. The calibration range was 0.09375–75 µg·mL^−1^ (r^2^ > 0.999); the between-standards standard deviation was <10% across the range (intra-assay). Both the samples and the column were maintained at room temperature. The %EE was calculated using Equation (1). Six independent batches were analyzed for %EE, and each batch was quantified from three independent prepared samples (triplicate sample preparations). The results are reported as mean ± SD.

#### 2.8.4. Ethanol Removal and Monitoring with At-Line Raman

After manufacture, the solvent was removed by magnetic stirring at room temperature. The residual ethanol was monitored at-line using Raman spectroscopy (Visum Raman In-Line™, IRIS Technology Solutions, Barcelona, Spain) at predefined time points to determine the end point (residual ethanol ≤ 1% *w*/*w*), which was periodically confirmed by gas chromatography (GC) (Section 2.8.5). Alternative membrane-based options (centrifugal diafiltration, dialysis, and tangential flow filtration) were explored during development but ultimately not used for the confirmation batches due to decreases in the apparent encapsulation efficiency observed in exploratory trials.

For safe and efficient measurements, IRIS designed a dedicated vial holder that immersed the probe in 7–12 mL vials. The holder is fully enclosed to prevent any laser leakage and was hard-wired to the instrument’s safety interlock so that the laser automatically disabled when the door was opened.

The calibration model was developed with 33 samples (0–10% *w*/*w* ethanol). Reference values were determined by GC on the same samples. PLS toolbox software (PLS_Toolbox, 2016, Eigenvector Research, Inc., Manson, WA, USA) running in Matlab (MATLAB, Version R2018a, The MathWorks Inc., Natick, MA, USA) was used to treat the spectra and optimize the calibration model. A 5-fold venetian blinds cross-validation was used to identify the optimal number of latent variables and to test model performance. The model development, cross-validation, and final fit statistics are described in the Results section.

For in-process control, two consecutive at-line Raman readings ≤ 1.0% *w*/*w* were required to declare the solvent removal end point. The Limits of Detection (LOD) and Limits of Quantification (LOQ) were estimated from low-ethanol standards and are reported in the Results.

#### 2.8.5. Determination of Ethanol by Gas Chromatography

Ethanol was quantified by GC and served as the orthogonal reference for Raman calibration and periodic verification. Analyses were performed on a gas chromatograph model 6890N (Agilent, Santa Clara, CA, USA) with a flame ionization detector, a split/splitless inlet, and an automatic injector 7683B. The target analyte was separated through a capillary column coated with fused silica (DB-624 30 m × 0.32 mm × 1.8 μm) (Agilent, Santa Clara, CA, USA). The oven temperature was programmed with a gradient, starting at 40 °C for 6 min and then raised to 220 °C at a rate of 15 °C/min (5 min hold). The temperatures of the injector and detector were 240 °C and 280 °C, respectively. Helium was used as a carrier gas at a flow rate of 2.1 mL/min and the analyses were performed in split mode (1:20). A 7:100 dilution of the sample was performed in dimethylsulfoxide, and the sample volume was 0.1 µL.

## 3. Results and Discussion

### 3.1. Work Temperature Determination

DSC was used to analyze the thermal behavior of phospholipids and cholesterol, aiming to determine their phase transition, to establish an optimal working temperature for incorporating these lipids into liposomal systems [29,30]. In DSC thermograms, the peak temperature (T_peak_) corresponds to the maximum heat flow of a transition and does not represent the end of the event, which is defined by the return to baseline (offset/endset). Transition temperatures are therefore reported as T_peak_ values as a consistent comparative descriptor across materials. The transition region was interpreted qualitatively to define the working window.

**Figure 2:**
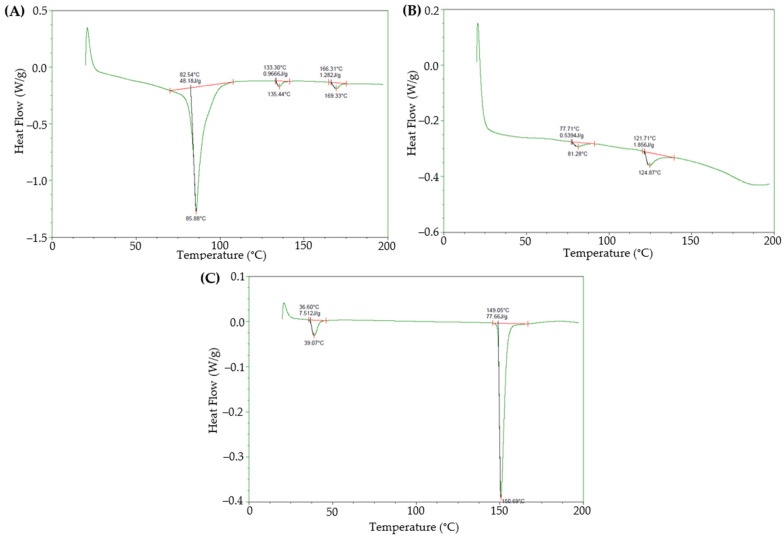
Representative thermograms for fresh material lots for (**A**) phosphatidylcholine hydrogenated (phospholipon 90H), (**B**) phosphatidylcholine (phospholipon 90G), and (**C**) cholesterol.

Phosphatidylcholines are materials with documented melting temperatures higher than 200 °C [31] and several thermal transitions to liquid crystalline states observed at lower temperatures [32,33]. Phosphatidylcholine hydrogenated has a main endothermic transition at 82.54 °C (48.18 J·g^−1^) and 85.88 °C (Figure 2A), associated with the gel-to-liquid crystalline phase change. This transition allows the lipid to attain the fluidity necessary to form flexible bilayers in liposome preparation [30]. Phosphatidylcholine presents a main phase transition at 77.71 °C (0.5394 J·g^−1^) and 81.28 °C (Figure 2B). Additionally, secondary peaks were observed at 121.71 °C and 124.87 °C (1.856 J·g^−1^), as previously reported [33], indicating possible minor structural reorganizations at higher temperatures. These secondary events are reported for completeness and were not used to define the processing temperature window, which was selected based on the main chain transition region relevant for bilayer fluidity during liposome formation. A distinct high-temperature melting event for phosphatidylcholine was not resolved within the DSC program used in this study (20–200 °C), which is consistent with literature reporting melting-like events above 200 °C [31] and with the fact that natural-mixture phosphatidylcholine grades may exhibit broadened or low-enthalpy high-temperature transitions.

This behavior suggests that the optimal working temperature for both phospholipids, to facilitate liposome formation, should be within the 70–85 °C range. At this temperature range, the lipids are in their liquid crystalline phase, allowing greater flexibility and stability in the lipid bilayer [29,30].

Cholesterol is a critical component in liposome stabilization, and its thermal behavior is fundamental for determining how it integrates into the lipid bilayer [30]. Its thermal analysis shows two relevant endothermic events: one at 36.60 °C (7.512 J·g^−1^), representing a secondary transition, likely a structural reordering, and another at 149.05 °C (77.66 J·g^−1^), identified as the cholesterol melting temperature. This corresponds to the complete solid-to-liquid phase transition, indicating the temperature at which cholesterol loses its crystalline structure (Figure 2C). This point is particularly important, as working at a higher temperature could compromise the stability of cholesterol and affect the integrity of the liposomes [34].

Based on these results, an optimal temperature of 70–85 °C was selected, just above the phase transition temperature, where the lipids are in a liquid crystalline state ideal for forming stable bilayers in liposomes. This range ensures that the lipids have the required fluidity without risking thermal decomposition. At this temperature, cholesterol can be inserted into the lipid bilayer, contributing to liposomal structural stability by restricting fatty acid chain mobility and enhancing bilayer rigidity.

DCF was not analyzed by DSC in this work; instead, its thermal stability under the selected processing conditions is discussed based on the literature data. DCF has been reported to decompose in the range of 270–390 °C, via initial melting followed by the decomposition process [35,36,37]. At temperatures below this threshold, therefore, of the order of 70 °C, for instance, DCF should remain stable, making this a suitable temperature for its incorporation.

### 3.2. Sonication: Lab-Scale Optimization and Scale-Up

#### 3.2.1. Lab-Scale Formulation Screening and Final Performance

A preliminary formulation screening was conducted using probe sonication as the reference manufacturing method to fix the lipid composition and solvents while varying (i) diclofenac loading (0.2–0.5% *w*/*v*) and (ii) surface-active excipients (Tween-80, benzalkonium chloride—BAK—or both). The working hypothesis was that the cationic BAK could enhance diclofenac association (anionic at pH 7.2–7.4). Under identical sonication conditions, all candidates produced sub-100 nm vesicles; however, PDI was lowest when Tween-80 was present and 0.5% (*w*/*v*) diclofenac was used. Mechanistically, low fractions of polysorbate-80 act as membrane “edge activators”, lowering bending elasticity and facilitating more uniform vesicle breakup/reassembly under ultrasound, which is consistent with the lower PDI observed here. At higher surfactant fractions, however, non-ionic surfactants can begin to solubilize bilayers into mixed micelles, so keeping Tween-80 at 0.05% *w*/*w* is prudent [38,39,40]. Based on size/PDI and practicality (avoidance of a cationic excipient), the Tween-80-only composition at 0.5% diclofenac was retained for subsequent work.

The placebo liposomes prepared under the same composition and processing framework showed a Z-average of 88.3 ± 3.7 nm, but a higher PDI of 0.275 ± 0.027 and a slightly less negative Z-potential −14 ± 0.3 mV, indicating broader size distributions in the absence of drug. In contrast, diclofenac-loaded liposomes produced with short sonication bursts (20% amplitude; 15 s) yielded Z-average 86.4 ± 8.4 nm, PDI 0.145 ± 0.026, %EE 88.8 ± 8.2%, and Z-potential −18 ± 0.4 mV. This modestly more negative zeta potential and narrower dispersity are consistent with partial interfacial localization/partitioning of anionic diclofenac at pH 7.2–7.4, which increases electrostatic repulsion and sharpens the distribution. Related biophysical work shows diclofenac perturbs phosphatidylcholine bilayers and alters interfacial charge and packing. By contrast, including BAK (a quaternary ammonium surfactant) introduces the possibility of ion pairing with anionic drugs, which can shift colloidal stability and promote heterogeneity; this pharmacotechnical behavior is well-recognized in formulation science and supports excluding BAK from the final composition [41]. At process level, ultrasonic cavitation reduces vesicle size by fragmenting larger lamellae into bilayer fragments that rapidly re-close as smaller liposomes, so cumulative energy delivery is a physically meaningful scale-up lever, a principle used later in our energy-normalized model [42]. When interpreting PDI, values < 0.20 denote narrow distributions, whereas very high PDI (>0.70) limits DLS interpretability. Moreover, PDI estimation becomes noisier at very low values, which should be considered when comparing close formulations [43]. Finally, although |Z-potential| ≈ 30 mV is a common heuristic for purely electrostatic stabilization, the combination of a modestly negative Z-potential with Tween-80-mediated steric effects is often sufficient for kinetic stability under our measurement conditions and for topical use [44]. These lab-scale dataset the acceptance targets for scale-up (Z-average ≤ 100 nm; PDI ≤ 0.30; %EE ≥ 80%).

#### 3.2.2. Sonication Scale-Up: Plackett–Burman Screening and Energy-Based Modeling

A class III Plackett–Burman screening design with eleven runs was carried out with placebo liposomes to map scale sensitivities. Amplitude (%), Batch Volume (L), Sonication Cycles, and Jacket Temperature (°C) were the process parameters selected, and the responses were Z-average (nm) and PDI (Table 3). Plackett–Burman designs are resolution III screens; thus, two-factor interactions are generally confounded with main effects. Estimates should be interpreted as directional to rank factors rather than as final quantitative models—hence, our subsequent confirmation by energy-based modeling [28,45].

Amplitude (%) and Batch Volume (L) were significant for Z-average (nm). Equation (2) shows the resulting main-effects model [28]:

Z-Average (nm) = 728.20 − 5.922 Amplitude (%) − 8.39 Batch Volume (L)(2)

As indicated by Equation (2), increasing Amplitude and Batch Volume decreased particle size, consistent with greater cavitation and improved hydrodynamics in the flow cell. The model adequacy was excellent (R^2^_adj_ = 97.57%, R^2^_pred_ = 96.47%), indicating that almost all size variability within the design space was captured by these two terms. The amplitude effect aligns with ultrasound-driven cavitation, which fragments larger lamellae into bilayer phospholipid fragments that re-close as smaller vesicles. Higher acoustic intensity increases transient bubble activity and shear at the probe tip, accelerating size reduction [42,46]. The negative coefficient for Batch Volume likely reflects flow cell hydrodynamics at the chosen fixed recirculation rate (2 L·min^−1^): larger volumes increase mean residence time per pass and smoothen local energy delivery, favoring more uniform breakup even though total energy per mass is controlled later via the energy model [28,47].

For PDI, Amplitude (%), Batch Volume (L), and Sonication Cycles were significant. The regression Equation (3) obtained was the following [28]:

PDI = 0.6267 − 0.000904 Amplitude (%) + 0.000586 Batch Volume (L) − 0.01817 Sonication Cycles(3)

This model showed good predictability (R^2^_adj_ = 86.33%, R^2^_pred_ = 77.62%). Higher Amplitude and more Sonication Cycles narrowed the size distribution (lower PDI), whereas larger Batch Volume slightly increased PDI, opposite to its effect on size [28].

The Amplitude (%) factor significantly affected both particle size and PDI. Increasing the sonotrode oscillation amplitude raises the cavitation energy, thereby yielding smaller particles with a narrower size distribution. The number of Sonication Cycles was also significant for PDI: each pass through the flow cell supplies an additional energy dose while allowing brief cooling/relaxation, which collectively reduces residual aggregates and sharpens the distribution. In contrast, Batch Volume showed a bidirectional effect: larger volumes tended to decrease size (more homogeneous recirculation and residence time distribution) but slightly increased PDI, suggesting broader local energy exposure despite jacketed temperature control. Importantly, because Plackett–Burman screens confound interactions, the Batch Volume term in the PDI model may proxy unmodeled interactions (e.g., Amplitude × Volume or Cooling Efficiency × Volume). This motivated switching to a single-variable energy density model to collapse Amplitude/Cycle/Volume effects into one physically meaningful CPP [28,45].

The proposed models describe the responses measured at the end of the sonication process; however, understanding the particle evolution during processing is also relevant. Moreover, the resulting equations included different sets of significant factors: the particle size was driven by two variables (Amplitude and Batch Volume), whereas PDI was described by three variables (Amplitude, Batch Volume, and Sonication Cycles). Among the factors assessed, Batch Volume showed the smallest effect on both particle size and PDI, while Amplitude and Sonication Cycles exhibited the strongest influence. This behavior is consistent with the cavitation energy (W·s) delivered to the formulation. Taken together, these findings motivated the evaluation of alternative models in which particle size and PDI are predicted as a function of cavitation energy normalized by Batch Volume. This single-factor model is simpler and more practical for scale-up applications. Cavitation (acoustic) energy has previously been identified as a critical variable in liposome production [48]. From a metrology perspective, reporting and controlling the delivered acoustic energy (or intensity) is recommended to improve reproducibility across instruments, since absolute “% Amplitude” settings depend on probe characteristics, horn geometry, and coupling conditions; expressing cumulative energy per unit volume (W·s·L^−1^) therefore provides a more transferable process metric [28,49].

Accordingly, aliquots were taken from the pre-mix (before sonication) and after each sonication cycle. For every aliquot, the cumulative energy recorded by the instrument was normalized by Batch Volume to obtain energy density (W·s·L^−1^), which was then related to the Z-average and PDI. This provides a practical scale-up handle; target size and dispersity can be achieved by meeting an energy density set point, independent of the specific combination of amplitude and cycle number. Prior studies likewise identify cavitation energy as critical in liposome production. Thermal management remains essential: processing within the liquid crystalline window of the lipids (Section 3.1) enables membrane fluidity during breakup/reformation while avoiding thermal degradation; because ultrasound induces localized heating, jacket control should be paired with short cycles to limit hot-spot accumulation [7,28,50].

**Figure 3:**
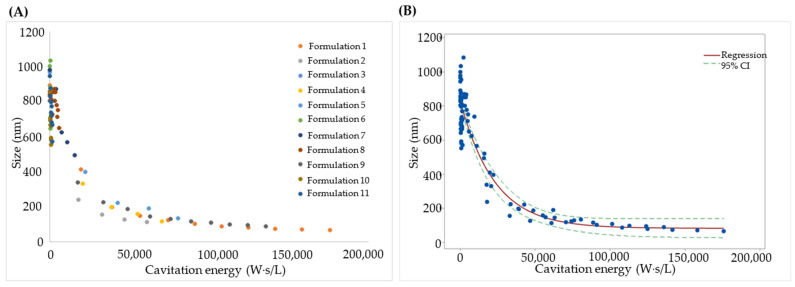
Size data for the scale-up formulations as a function of cavitation energy: (**A**) individual values for each batch, and (**B**) fit to a non-linear model. Blue dots represent experimental measurements; the solid line shows the regression fit and dashed lines indicate the 95% confidence interval. Reproduced with permission from Ref. [28].

Figure 3A shows the size values measured for each batch and the corresponding aliquots as a function of cavitation energy normalized by Batch Volume (W·s·L^−1^) [28].

The following regression equation (Equation (4)) was obtained using Minitab [28]:

Size (nm) = 83.29 + 742.12 e^−0.00044 × Energy^ (4)

where Energy represents the cavitation energy density (W·s·L^−1^). Figure 3B shows both the experimental size data and the non-linear regression fit with the 95% confidence interval (individual batch/aliquot values are reported in Figure 3A). The model achieved an adjusted R^2^ of 0.8999, indicating good agreement with the experimental data [28].

**Figure 4:**
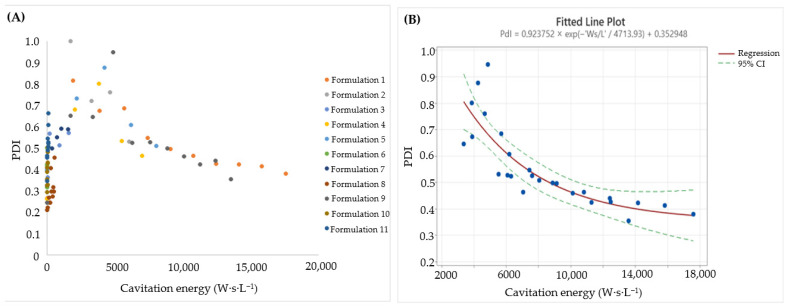
PDI data for the scale-up formulations as a function of cavitation energy: (**A**) individual values for each batch, and (**B**) fit to a non-linear model. Blue dots represent experimental measurements; the solid line shows the regression fit and dashed lines indicate the 95% confidence interval. Reproduced with permission from Ref. [28].

Figure 4A presents the PDI values measured for each batch and aliquot as a function of cavitation energy [28].

Because DLS algorithms are optimized for mono- or bimodal populations, PDI estimates are unreliable at very high polydispersity. This is evident in the initial region (0–4000 W·s·L^−1^), where the transient PDI increase does not reflect the longer-term effect of Sonication Cycles and Amplitude. Therefore, for the non-linear regression, we restricted the fit to energy densities > 4000 W·s·L^−1^ (beyond the point where the slope becomes negative), yielding a model that is more accurate and relevant for the final PDI of the manufactured formulation. This data-driven truncation is consistent with best practice for DLS interpretability (PDI >> 0.5), where inversion algorithms are unstable and trends can be misleading [28,43,44].

The resulting regression equation (Equation (5)) follows [28]:

PDI = 0.353 + 0.924 e^−4713.93 × Energy^ (5)

The empirical PDI data and the non-linear fit with 95% CI are presented in Figure 4B. The adjusted R^2^ of this model was 0.8379, indicating a good fit.

After obtaining the non-linear regression equations for size and PDI, different values of cavitation energy were simulated to observe the predicted values of both responses. As summarized in Table 4, achieving PDI < 0.5 and Z-average < 90 nm requires approximately 11,000 W·s·L^−1^. For an 8 L batch, this corresponds to a total sonication energy of 88,000 W·s. Practically, the energy density set point functions as a compact critical process parameter; by meeting ~11,000 W·s·L^−1^, comparable CQAs were obtained across the 8 L confirmation runs despite the differences in the instantaneous amplitude/cycle histories, supporting scale-invariant control [42,49].

Finally, an additional batch was produced and analyzed using the final configuration (100% amplitude, 11,000 W·s·L^−1^, 8 L batch volume), yielding an average diameter of 91.34 nm. The difference between the experimental diameter (91.34 nm) and the model-predicted Z-average (89.36 nm) at 11,000 W·s·L^−1^ corresponded to a bias of 2.22%. This low bias supports the accuracy of the model for predicting liposome Z-average within the defined design space [28,47].

#### 3.2.3. Pilot-Scale Confirmation Batches of Diclofenac-Loaded Liposomes

Two 8 L confirmation runs (Batches 12 and 13) were manufactured under the optimized sonication window (100% amplitude; target energy density ≈ 11,000 W·s·L^−1^; and jacket 30–45 °C; bulk maintained within the DSC-defined 70–85 °C range). Both batches were produced using the same formulation composition and the same target operating set points. The pre-mix was prepared as in Section 2.3, dissolving phospholipids, cholesterol, α-tocopherol, Tween 80, and sodium diclofenac in ethanol before the addition of the preheated aqueous phase. The dispersion was recirculated through the UIP1000HdT flow cell at 2 L·min^−1^ until the energy set point was reached.

**Figure 5:**
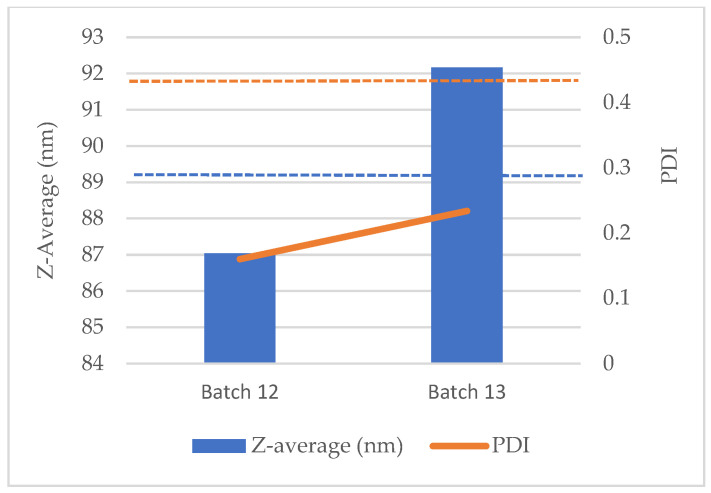
Observed vs. model-predicted size and PDI at 11,000 W·s·L^−1^. Bars represent the observed Z-average and the solid line represents the observed PDI; dashed horizontal lines denote the model predictions for Equations (4) and (5): 89.36 nm for Z-average and 0.443 for PDI.

For Batch 12, DLS at 25 °C (triplicates) yielded Z-average 87.04 nm and PDI 0.160; the zeta potential averaged −24.6 mV, and the encapsulation efficiency (%EE) by ultrafiltration–HPLC was 94.0%. For Batch 13, DLS gave Z-average 92.17 nm and PDI 0.234, and the zeta potential was −19.1 mV, with %EE 86.3%. The results are summarized in Table 5 and Figure 5. Across both batches, the outcomes fall within the predefined quality window (Z-average < 100 nm; PDI < 0.30) and align with the energy-based predictions at 11,000 W·s·L^−1^ (Equations (4) and (5); predicted Z-average 89.36 nm, PDI 0.443). The deviations from the size predictions were modest (−2.6% for Batch 12; +3.1% for Batch 13), and the observed PDIs were lower than the conservative model estimate, consistent with effective energy delivery and hydrodynamic control at scale. Taken together, these descriptive (n = 2) data document the reproducible attainment of the target operating window for DCF-loaded liposomes at the pilot scale.

The descriptive nature (n = 2) of these pilot runs is appropriate for process confirmation but does not constitute a process performance qualification (PPQ) dataset; regulatory frameworks typically expect three or more conformance batches for PPQ and long-run capability analysis (e.g., Cp/Cpk). Here, the intent is to verify the transferability of the energy density critical process parameter (CPP) established during screening to an 8 L scale—an approach aligned with Quality-by-Design principles that link CPPs to CQAs [51].

The slightly less negative zeta potential and higher PDI in Batch 13 are most plausibly attributable to routine run-to-run variability in acoustic energy distribution, residence time/temperature histories within the flow cell, and minor differences in interfacial conditions (e.g., buffer ionic strength or residual solvent), all of which can modulate bilayer packing and electrostatic screening; diclofenac–phosphatidylcholine systems are known to be sensitive to such interfacial/environmental factors. Nonetheless, the zeta potential values in the −19 to −25 mV range and PDI ≤ 0.234, together with Tween-80-mediated steric stabilization, are consistent with kinetic stability under the chosen measurement conditions [44].

Maintaining the bulk temperature within the DSC-defined liquid crystalline window (70–85 °C) during sonication likely facilitated membrane fluidity and efficient breakup/reformation while avoiding cholesterol/lipid degradation, complementing the energy density control of acoustic input [7,42].

The observation that PDIs were below the model’s conservative prediction is coherent with our modeling strategy; the PDI fit excluded the high-polydispersity, low-energy region (>0.5 PDI; Energy < 4000 W·s·L^−1^) to improve relevance for final product states. Consequently, the final model tends to overestimate dispersion at the production set point, which is a desirable bias from a control perspective [44].

Overall, these independent manufacturing batches corroborate that meeting the energy density set point reproduces the target CQAs at pilot scale, despite different instantaneous amplitude/cycle histories, supporting scale-invariant control of the sonication process [49].

### 3.3. Microfluidics: Effect of Flow Rate Ratio (FRR) and Total Flow Rate (TFR) on Liposome Physicochemical Attributes

Liposomes were prepared by microfluidics (Section 2.5) while varying the flow rate ratio (FRR, aqueous:solvent ratio) between 3:1 and 7:1 and the total flow rate (TFR) between 4 and 8 mL·min^−1^. The resulting Z-average, PDI, and %EE are summarized in Table 6 (n = 13). A clear dependency on FRR emerged: higher FRR systematically yielded smaller vesicles with low PDI and high %EE [6,10,52,53]. At FRR 7:1, sizes clustered at ~34–36 nm (PDI 0.068–0.127; %EE ≈ 92%), whereas FRR 5:1 produced ~51–52 nm liposomes with very low PDI (0.026–0.038) at similarly high %EE. In contrast, FRR 3:1 gave larger particles (~54–64 nm). Increasing TFR to 8 mL·min^−1^ partially compensated for this effect at low FRR, reducing size and narrowing dispersity. This is consistent with faster mixing and shorter micromixing times in the toroidal/hydrodynamic focusing regime; a well-established FRR/TFR–size relationship is documented for liposomes and lipid nanoparticles (LNPs) [10,52,53]. This benefit at low FRR/high TFR was accompanied by a modest drop in %EE for some runs (to ~83%), indicating a throughput–loading trade-off when dilution and mixing accelerate self-assembly beyond the optimal regime for diclofenac entrapment. Microfluidic rapid mixing can reduce particle size at the expense of cargo loading, depending on drug–lipid partitioning and solvent removal kinetics [26,52,53].

A screening regression (Minitab; α = 0.05) quantified the effects of FRR (X_1_) and TFR (X_2_) on the three responses (models shown in Table 7). The size model showed excellent explanatory power, capturing strong main effects and a significant FRR × TFR interaction. The size benefit of higher TFR is greater at low FRR, again in line with microfluidic solvent displacement theory [6,10,52,53,54]. The %EE model also fits well, recovering the observed efficiency decrease at FRR 3:1/TFR 8 mL·min^−1^ [52,55]. As expected for narrow PDI ranges, the PDI model displayed modest explanatory power, which is consistent with reports that device-specific hydrodynamics (mixer geometry, priming/wetting history) and subtle thermal drifts can drive residual variability in PDI even when FRR/TFR are controlled [6,9,26,56]. Taken together, the dataset delineates a practical operating window in which FRR 7:1 combined with TFR 6–8 mL·min^−1^ robustly delivers sub-40 nm liposomes with PDI ≤ 0.13 and %EE ≈ 92%, while operation at lower FRR may require balancing throughput gains against %EE losses [53,54,56].

To jointly balance size, dispersity, and loading, a multi-response desirability optimization (Minitab) was run with the following goals: minimize PDI (upper bound 0.20), target size of 75 nm (acceptable 50–100 nm), and maximize %EE (lower bound 85%). The recommended set point was FRR = 3:1 and TFR = 4 mL·min^−1^, with predicted PDI = 0.0859 (SE 0.0189; 95% CI 0.0430–0.1287; and 95% PI 0.0058–0.1659), size = 62.27 nm (SE 2.00; 95% CI 57.75–66.78; and 95% PI 53.83–70.70), and %EE = 93.20% (SE 1.18; 95% CI 90.52–95.87; and 95% PI 88.19–98.20), yielding a composite desirability of 0.560 (Table 8). This “balanced” optimum positions the formulation in the 60–70 nm range with first-decimal PDI and high %EE. If the priority is minimum size, the model shifts the optimum toward higher FRR (7:1) and moderate–high TFR (6–8 mL·min^−1^) to obtain <40 nm, at the expense of the 75 nm target [1,2,3,4]. This behavior mirrors the canonical FRR-up/TFR-up → size-down trend reported for both liposomes and LNPs, including on the NanoAssemblr^®^ platform (hydrodynamic/bifurcating mixers) [56].

Under this window, the confirmation batches (FRR 3:1; TFR 4 mL·min^−1^, batch IDs: 27, 28, and 29) yielded Z-average 63.64 ± 2.35 nm, PDI 0.1330 ± 0.0115, %EE 92.67 ± 2.52% (n = 3), and the zeta potential −29.35 ± 0.92 mV (n = 3). As summarized in Table 9, the model–experiment bias was small for size and %EE (+2.20% and −0.57%, respectively), confirming good predictivity (the observed means lie within the model 95% CI). The PDI showed a larger relative bias (+54.65%), but this stems from the very low theoretical value (0.086); the absolute deviation is modest (≈+0.047) and remains well below the specification limit (PDI < 0.20). Such PDI scatter at low absolute values is consistent with the known sensitivity limits of DLS algorithms and minor thermohydrodynamic fluctuations; additionally, the PDI regression displayed the lowest explanatory power among the three responses (R^2^ = 0.483; R^2^_adj_ = 0.311), as shown in Table 7, so larger residuals at confirmation are expected [56].

The placebo prepared by microfluidics (Batch 27) did not form a nanosized dispersion (Z-average 489.4 nm; PDI 0.601). This is mechanistically plausible: removing diclofenac eliminates an anionic co-solute that can contribute to interfacial charge/curvature stabilization and transiently increase negative zeta potential. Without that contribution, the FRR/TFR window optimized for the drug-loaded system becomes sub-optimal for a neutral placebo, shifting packing toward multilamellar/aggregated structures that manifest as high PDI. Moreover, cargo can influence nucleation–growth pathways under rapid mixing, so placebos may require the re-optimization of FRR/TFR and/or minor changes to ionic strength, lipid concentration, or the inclusion of charge donors to restore colloidal stability despite Tween-80 steric effects [6,52,55,57].

### 3.4. Microreactor Scale-Up

Liposomes were produced using a proprietary high-turbulence microreactor (MyBiotech GmbH) operated with two HPLC pumps (Section 2.7). The organic and aqueous phases were prepared with the same composition used throughout this study. Both streams were preheated and combined at controlled FRR (aqueous:organic ratio) between 10:1 and 4:1, exploring TFR in the 46–50 mL·min^−1^ range. At FRR 10:1, the resulting dispersions did not meet the predefined quality criteria. Progressively increasing the organic-phase fraction led to systematic improvements in both size and dispersity, with the best outcome achieved at FRR 4:1 and TFR 50 mL·min^−1^ (Table 10). Under these conditions, for Batch 32, the Z-average reached ~50 nm with PDI 0.254 while %EE remained high (~95%), indicating efficient drug retention despite the higher throughput characteristic of the microreactor setup. The observed trend, smaller size and lower PDI at a lower FRR (i.e., higher solvent fraction), is consistent with rapid solvent–antisolvent micromixing that accelerates nucleation and truncates growth, a well-described outcome in impinging/turbulent jet mixers and flash nanoprecipitation analogs [15,58]. Within the narrow TFR band tested (46–50 mL·min^−1^), this FRR-dependence suggests that mixing time, rather than absolute residence time, was the dominant lever; in turbulent/impinging microreactors, shortening the micromixing time scales with increasing flow and momentum flux, yields finer dispersions [58,59].

Mechanistically, the microreactor outcome aligns with microfluidic solvent displacement theory, where particle size decreases as the effective dilution rate and local supersaturation rise, but here this is achieved at an order-of-magnitude higher throughput than was the case for bench microfluidics (50 vs. 4–8 mL·min^−1^). Such “microfluidic-like” quality at an elevated flow is consistent with confined impinging/coaxial turbulent mixers that deliver sub-100 nm nanoparticles at kg·day^−1^ scale while maintaining tight size control [58]. Recent liposome-specific work using ultrasonic/impinging microreactors likewise reports FRR- and flow-governed reductions in size and dispersity, supporting the generality of the mixing–quality relationship observed here [60].

The limitations of the present dataset include the small number of conditions evaluated (n = 3) and the proprietary nature of the reactor (no internal geometry disclosed). Future work should expand the experimental matrix (e.g., lipid concentration, temperature, and post-processing) and include additional replicates to develop a predictive model akin to the energy-based framework used for sonication. Nonetheless, the current results establish a feasible operating point (FRR 4:1; TFR 50 mL·min^−1^) that delivers ~50 nm, PDI ~0.25, and %EE ~95%, providing a practical basis for further scale-up. Because mixing intensity in impinging/turbulent reactors increases with flow, we hypothesize that quality gains (smaller size/narrower PDI) are achievable at higher TFR—subject to pressure drop and thermal management constraints—mirroring the scale-up guidance from micromixing theory [59].

### 3.5. Post-Processing Control: Ethanol Removal and Monitoring (Raman vs. GC)

**Figure 6:**
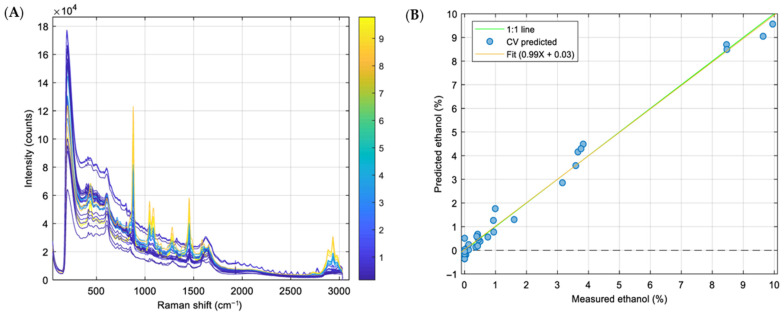
(**A**). Raman spectra of the samples used to develop the ethanol calibration model colored by ethanol concentration. (**B**). Predicted versus measured regression line for the ethanol model.

A Raman calibration model was developed to monitor ethanol during solvent removal. The spectra of the 33 calibration samples (0–10% *w*/*w* ethanol) showed bands that increased monotonically with ethanol content and matched literature assignments for EtOH (Figure 6A) [61]. Optimal performance was obtained by modeling the 800–1500 cm^−1^ region, which contains the most informative ethanol bands; baseline noise attributable to liposome light-scattering was effectively corrected using a first-derivative pre-treatment. The predicted versus the measured plots were close to the ideal 1:1 line (Figure 6B), with R^2^ for calibration and 5-fold venetian blinds cross-validation both being ≈0.99 and having low RMSEs (0.27% and 0.32% *w*/*w*, respectively), indicating the absence of overfitting. Blank samples returned values between −0.1% and 0.1% *w*/*w*; therefore, 0.1% *w*/*w* was adopted as the practical detection limit, and values < 0.1% were treated as ethanol-free in routine decisions.

**Figure 7:**
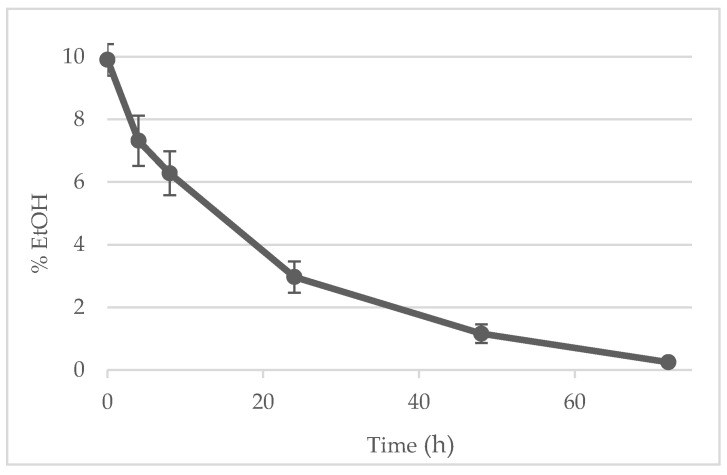
Ethanol content in liposomes formulation during evaporation.

Membrane-based solvent exchange (dialysis and centrifugal diafiltration) efficiently reduced ethanol (diafiltration reached the target after ~four diafiltration volumes), but the final formulations exhibited an ~85% decrease in diclofenac relative to the starting content, indicating drug loss to the retentate/filtrate during solvent removal. Consequently, for the confirmation batches, we adopted simple magnetic stirring as the primary ethanol removal method and monitored the process at-line by Raman. A representative time course showed a monotonic decline to <1% within 6 h (Figure 7), at which point the Raman end point criterion was met. Periodic GC measurements confirmed the Raman-based end point classification.

Taken together, these data show that (i) the Raman model provides an accurate, rapid (results in seconds), non-destructive quantification of ethanol in liposomal matrices for in-process control, and (ii) direct evaporation by magnetic stirring removes ethanol to specification without the diclofenac losses observed when using membrane-based solvent exchange. Compared with gas chromatography, at-line Raman avoids time-consuming injections and oven programs (minutes per run), preserves the sample for further tests, and can be deployed directly at the process line, reducing analytical turnaround and enabling true PAT/real-time decision making. Periodic GC checks were retained as an orthogonal reference method.

### 3.6. Cross-Method Comparison of Physicochemical Attributes at Optimal Conditions

To compare the three manufacturing routes on a comparable basis, we benchmarked each method at the best-performing window identified in this study and collated the CQAs—Z-average, PDI, %EE, and zeta potential—together with an indication of practical throughput/scale (Table 11).

Under sonication, lab-scale production (UP400st, 30 mL; Section 2.3) at 20% amplitude for 15 s (≈2000 Ws per batch) yielded DCF-loaded liposomes with Z-averages of 86.4 ± 8.4 nm and PDI 0.145 ± 0.026, a domain appropriate for topical products. Scaling to 8 L in a flow cell configuration (UIP1000HdT) while controlling via energy density (11,000 W·s·L^−1^ at 100% amplitude) reproduced this quality envelope: two independent pilot batches delivered 87.04/92.17 nm, PDI 0.160/0.234, %EE 94.0/86.3%, and zeta potential −24.6/−19.1 mV (Section 3.2). The small lab → pilot shift in mean size (<5%) and comparable dispersity indicate that meeting the cumulative cavitation energy set point is an effective, scale-invariant control lever, achieving similar CQAs despite the change in scale and hydrodynamics. This is consistent with the established link between ultrasound intensity/cavitation and vesicle downsizing, and with guidance on energy-normalized acoustic processing [42].

Microfluidics, implemented on the NanoAssemblr^®^ platform operated at the desirability-based set point FRR 3:1; TFR 4 mL·min^−1^, produced smaller and more uniform vesicles: Z-average 63.64 ± 2.35 nm, PDI 0.1330 ± 0.0115, %EE 92.67 ± 2.52%, and zeta potential −29.35 ± 0.92 mV, in agreement with the model predictions (Section 3.3). When minimum size is prioritized over the balanced target, microfluidics can push below 40 nm by operating at FRR 7:1 with TFR 6–8 mL·min^−1^, maintaining low PDI and high %EE at bench-scale throughput. The NanoAssemblr^®^ platform also includes larger-scale instruments (e.g., Blaze/GMP systems) that facilitate straightforward process translation and scale-up [26]. These trends align with hydrodynamic-focusing/solvent displacement theory, where FRR and TFR govern dilution and micromixing times, enabling predictable size control and scalable translation [6,10,52].

The high-turbulence microreactor provided the best quality/throughput compromise. At FRR 4:1 and TFR 50 mL·min^−1^, it achieved Z-average 50.1 ± 3.6 nm, PDI 0.254 ± 0.041, %EE 94.7 ± 1.4%, and a zeta potential of −9.8 ± 0.4 (Section 3.4), i.e., microfluidic-like sizes with ~10× higher flow. Within the explored window, decreasing FRR (higher organic-phase fraction) improved size and dispersity without compromising %EE, consistent with intensified micromixing. Notably, the microreactor operated at 50 mL·min^−1^ in this study; this flow was selected because it matched the upper delivery capacity of the small HPLC pumps available and the limited sample volume—indeed, ~50 mL·min^−1^ is near the lower end of the usual operating range for these systems, which commonly run at higher TFRs. Because its micromixing intensity increases with flow, exploring higher TFRs is likely to shift the microreactor further toward microfluidic-like sizes while preserving its throughput advantage. This is consistent with confined impinging/turbulent mixers and continuous-flow liposome reactors, where momentum-driven micromixing is the dominant lever for nanoparticle size at production-relevant throughputs [15,58,62].

From an application standpoint, the “optimal” liposome size is treatment modality dependent. For diclofenac, the primary intended use is local/topical anti-inflammatory delivery, where vesicles in the ~50–100 nm range are commonly targeted to balance dispersion uniformity, kinetic stability, and distribution within superficial/appendageal pathways (e.g., hair follicles), while still favoring local deposition at the application site. Within this topical context, sizes closer to ~80–100 nm (as achieved by sonication at lab and pilot scale) are widely used and can be advantageous when robustness and site retention are prioritized. In contrast, smaller vesicles in the ~50–70 nm domain (as achieved by microfluidics and the high-turbulence microreactor under the selected conditions) may be preferred when a smaller size is desired, for instance to support more homogeneous dispersions and distribution within superficial pathways. Therefore, rather than a single “best” size, the target size should be selected based on the intended route and depth of action, and then matched to the manufacturing method that most reliably delivers that size window [5,19,20,21,47].

At fixed nominal drug loading, liposome size does not change the total diclofenac added to the formulation, but can affect how diclofenac is distributed between liposome-associated and free fractions. In this study, the total diclofenac dose is set by the formulation composition (0.5% *w*/*w* sodium diclofenac in the final batch). However, changes in vesicle size (and the associated specific surface area) can influence drug partitioning between the bilayer/interface and the aqueous phase, which is captured experimentally by the drug association metrics (operationally reported here as %EE). Smaller vesicles provide a higher interfacial area per unit lipid mass and may favor a larger fraction of drugs associated with the lipid bilayer for amphiphilic/partially interfacial drugs, whereas larger vesicles may shift the balance depending on drug–lipid affinity and aqueous solubility. Consequently, for a constant total drug input, size differences primarily affect the free-drug fraction in the continuous phase and the liposome-associated fraction, rather than the nominal total diclofenac present. In our study, despite size differences across methods (~50–90 nm), %EE remained high (~86–95%), indicating that within this size domain the manufacturing route did not materially compromise diclofenac association under the selected composition and post-processing conditions.

When translating these findings into a choice of process, priorities matter. If your primary goal is minimum size and narrow PDI (e.g., <60–70 nm, PDI ≤ 0.15) at the bench scale or during formulation screening, microfluidics is the most direct route: quality can be controlled through FRR/TFR, accepting lower per-device throughput, and retaining a clear scale-out path via platform hardware. If sub-100 nm with acceptable PDI for topical use is required along with low initial investment and operational simplicity, sonication is attractive, governing the process by energy density (W·s·L^−1^) and mitigating heat effects with short cycles. Where throughput and scale-up are the dominant constraints and microfluidic-like sizes are still desired, a turbulent/impinging microreactor offers the best quality/throughput balance. Here FRR may be tuned at elevated TFR, mapping the operable window (pressure/temperature). Across routes, %EE remains high (~86–95%) and employing Z-pot ≈ −19 to −30 mV with Tween-80 supports kinetic stability. Consequently, selection can be driven by target size/PDI, required throughput, facility/equipment footprint, PAT readiness, and scale strategy rather than physicochemical feasibility alone [44].

### 3.7. Cross-Process Morphology by Cryo-TEM

**Figure 8:**
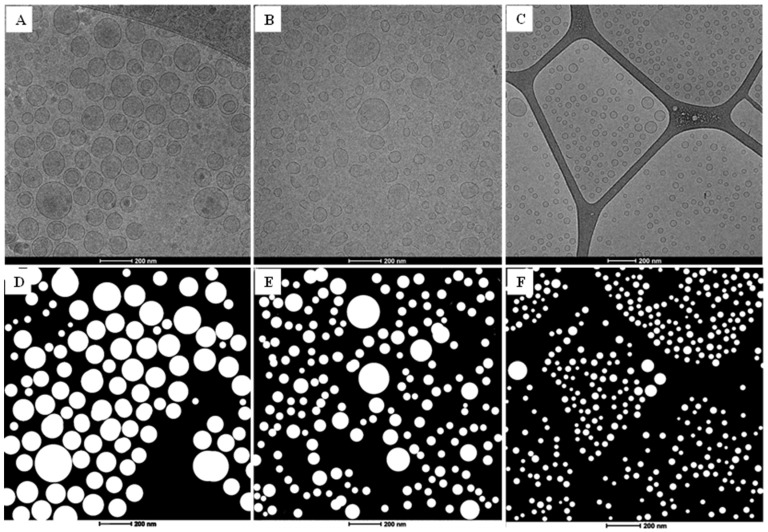
(**A**–**C**) are representative Cryo-TEM images of loaded diclofenac formulated by the different proposed methods: sonication (Batch 13), microfluidics (Batch 28), and microreactor (Batch 32), respectively. The captures show well-defined liposomal structures with a spherical morphology, monolamellarity, and a generally uniform size. (**D**–**F**) are the Image J image analysis obtained selecting all the nanoparticles from images (**A**–**C**).

Cryo-TEM is currently one of the most informative microscopy techniques for liposome characterization, as it circumvents artifacts associated with chemical fixation, dehydration, sectioning, and staining, all of which can alter liposome morphology [63,64,65]. Cryo-TEM imaging was performed on representative batches produced at the selected set points: Batch 13 (sonication), Batch 28 (microfluidics), and Batch 32 (microreactor). The images obtained (Figure 8) allow a direct evaluation of the morphology and size of diclofenac liposomes produced by the different fabrication methods: microfluidics, sonication, and microreactor (Table 12).

The physicochemical characterization of diclofenac-loaded liposomes by dynamic light scattering (DLS) and cryo-TEM revealed clear differences in size and polydispersity depending on the production method (Table 12). Since DLS and cryo-TEM interrogate different physical states and weighting schemes—DLS reports intensity-weighted hydrodynamic diameters in dispersion (including the hydration layer), whereas cryo-TEM provides number-weighted geometric diameters of vitrified particles—systematic offsets in size and PDI are expected. Despite this, both techniques yielded values of the same order and coherent trends across processes.

Liposomes obtained by sonication showed the largest diameters, with DLS giving an average size of 86.0 ± 1.1 nm and a PDI of 0.258 ± 0.01. Cryo-TEM analysis of 107 particles confirmed slightly larger structures (94.6 nm on average) and a somewhat lower PDI (0.205), indicating relatively uniform but consistently large vesicles.

The liposomes produced via microfluidics were smaller, with DLS values of 63.6 ± 2.4 nm and PDI of 0.133 ± 0.01. The cryo-TEM analysis of 183 particles showed comparable sizes (59.9 nm on average), though with a higher PDI (0.240), suggesting a broader distribution of particle diameters despite the smaller mean size.

Finally, the microreactor-derived liposomes displayed the smallest diameters. DLS returned a mean size of 50.1 nm with a PDI of 0.254, whereas the cryo-TEM analysis of 393 particles indicated even smaller sizes (38.2 nm) and the lowest heterogeneity (PDI 0.066). This highlights the capacity of the microreactor method to produce highly uniform liposomes with significantly reduced dimensions compared to sonication and microfluidics.

Overall, both DLS and cryo-TEM results followed the same trend in liposome size and distribution: sonication > microfluidics > high-turbulence microreactors, confirming the strong influence of the fabrication method on the resultant liposome dimensions. The slight differences between techniques are consistent with the hydration layer measured by DLS, which typically leads to larger apparent diameters compared to direct imaging by cryo-TEM and with the intensity vs. number weighting inherent to each method [65].

## 4. Conclusions

This study establishes a practical, QbD-aligned framework to manufacture diclofenac-loaded liposomes by three complementary methods. Probe sonication can be scaled reliably by controlling a single, physically grounded metric energy density (11,000 W·s·L^−1^) reproducing lab-scale quality at 8L. Microfluidics provides the finest size control via FRR/TFR tuning (balanced set point FRR 3:1, TFR 4 mL·min^−1^), while a high-turbulence microreactor achieves microfluidic-like sizes (~50 nm) at higher throughput (50 mL·min^−1^), highlighting a quality-throughput trade-off. Across methods, target CQAs for topical use (≈50–100 nm, PDI ≤ 0.30, high %EE) were met and corroborated by cryo-TEM. The target size window should be selected according to the intended use (e.g., topical local retention vs. preference for smaller vesicles), and the manufacturing route can then be chosen accordingly. Post-processing was streamlined with at-line Raman (calibrated to GC), enabling rapid, non-destructive control of residual ethanol. The method selection can therefore be guided by desired particle size/dispersity and operational constraints: sonication for simplicity and energy-based scale-up, microfluidics for tightest size control (via FRR/TFR), and microreactor for the advantages of high throughput and continuous production (via FRR at elevated TFR). Further work should address longer-term formulation performance, including storage stability, detailed drug–lipid association, and release behavior. Future work should also quantify the process yield using a predefined “nanoparticle yield” metric (e.g., mass balance or nanosized fraction yield after a defined separation step) to enable a complete comparison across routes and scales. Ultimately, all three routes met CQA targets for topical diclofenac.

## Figures and Tables

**Table 1:** Plackett–Burman screening matrix for sonication scale-up: factor settings for the 11 experimental runs. Reproduced with permission from Ref. [28].

Run	Amplitude (%)	Batch Volume (L)	Sonication Cycles	Jacket Temperature (°C)
1	100	8	10	30
2	100	8	4	45
3	20	8	4	30
4	100	1	4	30
5	100	1	4	45
6	20	1	4	30
7	20	8	4	45
8	20	1	10	30
9	100	1	10	45
10	20	1	10	45
11	20	8	10	45

**Table 2:** Factor levels evaluated in the screening design.

Factor	Lower Level	Higher Level
FRR	3:1	7:1
TFR	4	8

**Table 3:** Scale-up screening design: factors and responses for each liposome batch. Reproduced with permission from Ref. [28].

Batch	Amplitude (%)	Batch Size (L)	Sonication Cycles	Jacket Temperature (°C)	Z-Average (nm)	PDI
1	100	8	10	30	67.11	0.380
2	100	8	4	45	114.00	0.528
3	20	8	4	30	521.70	0.571
4	100	1	4	30	118.00	0.463
5	100	1	4	45	134.30	0.508
6	20	1	4	30	643.40	0.500
7	20	8	4	45	494.10	0.587
8	20	1	10	30	650.30	0.453
9	100	1	10	45	88.96	0.354
10	20	1	10	45	551.90	0.429
11	20	8	10	45	570.50	0.507

Note: Z-average (nm) and PDI values are means of three measurements (see Section 2.8.1).

**Table 4:** Prediction of liposome size and PDI as a function of sonication energy per liter of formulation. Reproduced with permission from Ref. [28].

Sonication Energy (W·s·L^−1^)	Predicted PDI	Predicted Size (nm)
1000	1.100	562.68
2000	0.958	392.96
3000	0.842	283.33
4000	0.749	212.51
5000	0.673	166.76
6000	0.612	137.21
7000	0.562	118.12
8000	0.522	105.79
9000	0.490	97.83
10,000	0.464	92.68
11,000	0.443	89.36

**Table 5:** Pilot-scale confirmation batches (8 L) with encapsulated diclofenac.

Batch	Z-Average (nm)	PDI	Zeta Potential (mV)	%EE
12	87.04	0.160	−24.6	94.0
13	92.17	0.234	−19.1	86.3

Note: Z-average (nm) and PDI values are means of three measurements (see Section 2.8.1). %EE values are means of three independently prepared samples per batch quantified by ultrafiltration-HPLC (see Section 2.8.3).

**Table 6:** Influence of flow rate ratio (FRR) and total flow rate (TFR) on Z-average, PDI, and %EE for microfluidic liposomes.

Batch	FRR	TFR (mL/min)	Z-Ave (nm)	PDI	%EE
14	7:1	6	35.03	0.076	91.89
15	3:1	6	53.99	0.075	92.07
16	3:1	4	61.01	0.120	92.50
17	7:1	4	33.90	0.127	92.23
18	3:1	8	57.46	0.037	82.99
19	7:1	8	35.85	0.068	92.10
20	5:1	4	51.40	0.038	92.17
21	7:1	4	33.90	0.127	92.23
22	3:1	4	64.36	0.084	92.31
23	7:1	8	35.85	0.068	92.10
24	5:1	8	51.92	0.035	92.31
25	5:1	6	51.44	0.026	92.15
26	3:1	8	58.95	0.038	83.50

Note: Z-average (nm) and PDI values are means of three measurements (see Section 2.8.1). %EE values are means of three independently prepared samples per batch quantified by ultrafiltration-HPLC (see Section 2.8.3).

**Table 7:** Screening models for microfluidic liposomes. Effects of flow rate ratio FRR (X_1_: 3, 5, and 7; aqueous:solvent) and total flow rate TFR (X_2_: 4, 6, and 8 mL/min) on size (Y_1_, nm), PDI (Y_2_), and encapsulation efficiency (Y_3_, %). Only terms significant at α = 0.05 were retained. Model coefficients, R^2^, and R^2^_adj_ are reported.

Quadratic Polynomial Model Equation	R^2^	Adj R^2^
Y_1_ = −91.81 − 8.47 X_1_ − 2.24 X_2_ + 0.402 X_1_ X_2_	0.9436	0.9089
Y_2_ = 0.1280 + 0.0035 X_1_ − 0.0142 X_2_ + 0.00034 X_1_ X_2_	0.4834	0.3112
Y_3_ = 108.98 − 2.53 X_1_ − 3.744 X_2_ + 0.564 X_1_ X_2_	0.7685	0.6913

X_1_: FRR; X_2_: TFR; Y_1_: hydrodynamic size; Y_2_: polydispersity index; and Y_3_: encapsulation efficiency.

**Table 8:** Multi-response desirability optimization at the selected set point (FRR 3:1; TFR 4 mL·min^−1^) for diclofenac-loaded microfluidic liposomes.

Size (nm) Fit	PDI Fit	%EE Fit	Composite Desirability
62.27	0.086	93.20	0.560

**Table 9:** Model prediction versus experimental confirmation at the set point (FRR 3:1; TFR 4 mL/min).

Property	Theoretical Value (95% CI)	Experimental Formulations	Bias (%)
Size (nm)	62.27 (57.75; 66.78)	63.64 ± 2.350	2.20
PDI	0.086 (0.043; 0.129)	0.133 ± 0.0115	54.65
%EE	93.20 (90.52; 95.87)	92.67 ± 2.52	0.57

**Table 10:** Influence of the manufacturing process parameters on the physico-chemical characteristics of liposomes during scale-up trials using microreactor technology.

Batch	FRR	TFR (mL/min)	Z-Ave (nm)	PDI	%EE
30	7:1	46	61.39	0.484	94.9
31	5:1	48	53.59	0.400	95.2
32	4:1	50	50.10	0.254	94.7

Note: Z-average (nm) and PDI values are means of three measurements (see Section 2.8.1). %EE values are means of three independently prepared samples per batch quantified by ultrafiltration-HPLC (see Section 2.8.3).

**Table 11:** Comparative performance of fabrication methods for DCF-loaded liposomes (CQAs and throughput).

Method/Scale (Set Point)	Z-Average (nm)	PDI	%EE (%)	Z-Potential (mV)	Throughput/Scale
Sonication—Lab (20% amplitude; 15; ~2000 W·s)	86.4 ± 8.4	0.145 ± 0.026	88.8 ± 8.2	−18.0 ± 0.4	30 mL per batch
Sonication—Pilot (energy density ≈ 11,000 W·s·L^−1^; 100% amplitude)	87.0/92.2	0.160/0.234	94.0/86.3	−24.6/−19.1	8 L (recirculation 2 L·min^−1^)
Microfluidics—Bench (FRR 3:1; TFR 4 mL/min)	63.6 ± 2.4	0.133 ± 0.012	92.7 ± 2.5	−29.4 ± 0.9	4 mL/min
Microreactor—Continuous (FRR 4:1; TFR 50 mL·min^−1^)	50.1 ± 3.6	0.254 ± 0.041	94.7 ± 1.4	−9.8 ± 0.4	50 mL/min

Note: Values are shown as mean ± SD; for sonication-pilot entries, two independent batches are shown as A/B. Z-average and PDI correspond to n = ten independent batches for sonication, n = five for microfluidics, and n = three for the microreactor; each batch value is the mean of three measurements (Section 2.8.1). Zeta potential values correspond to n = three independent batches for each method (Section 2.8.1). Encapsulation efficiency (%EE) values correspond to n = five independent batches for each method, quantified by ultrafiltration–HPLC; for each batch, %EE is the mean of three independently prepared samples (Section 2.8.3).

**Table 12:** Size and PDI comparison for the diferent formulation methods and measuring techniques.

Fabrication Method	DLS		Cryo-TEM		
	Z-Average (nm)	PDI	N° of Analyzed Particles	Diameter (nm)	PDI
Sonication	86.00 ± 1.09	0.258 ± 0.01	107	94.59 ± 42.87	0.205
Microfluidics	63.64 ± 2.35	0.1330 ± 0.01	183	59.85 ± 29.34	0.240
Microreactor	50.10 ± 3.60	0.254 ± 0.041	393	38.18 ± 9.78	0.066

Note: DLS values are reported as mean ± SD of independent batches (sonication n = ten; microfluidics n = five; microreactor n = three), with each batch value obtained as the mean of three measurements (Section 2.8.1). Cryo-TEM analysis was perfomed on Batch 13 (sonication), Batch 28 (microfluidics), and Batch 32 (microreactor). Cryo-TEM diameters are number-weighted and were obtained by ImageJ (version 1.54g) analysis; N indicates the number of analyzed particles. The dispersion metric reported focryo-TEMEM is calculated from the diameter distribution (see Section 3.7).

## Data Availability

Data is available upon request due to intellectual property.
